# Genes and the Environment in Cancer: Focus on Environmentally Induced DNA Methylation Changes

**DOI:** 10.3390/cancers15041019

**Published:** 2023-02-06

**Authors:** Fabio Coppedè

**Affiliations:** 1 Department of Translational Research and of New Surgical and Medical Technologies, University of Pisa, 56126 Pisa, Italy; fabio.coppede@med.unipi.it; Tel.: +39-050-2218544; 2 Interdepartmental Research Center of Biology and Pathology of Aging, University of Pisa, 56126 Pisa, Italy

Cancer has traditionally been viewed as a genetic disorder resulting from the accumulation of gene mutations, chromosomal rearrangements, and aneuploidies in somatic cells. Indeed, for several years cancer-related environmental factors have been tested for their potential to induce mutations of proto-oncogenes and tumor suppressor genes, as well as chromosome damage and mis-segregation, with non-mutagenic carcinogens also being observed [1,2,3,4]. Only since the end of the last century has it become increasingly evident that epigenetic modifications also play an important role in carcinogenesis [5]. Epigenetic modifications, including DNA methylation and post-translational modifications of histone tails, result in heritable changes in chromatin structure and gene expression that can be passed over several cell divisions, with functional consequences that are equivalent to those induced by gain-of-function or loss-of-function mutations [6]. Among them, DNA methylation represents one of the most investigated epigenetic marks in cancer, leading to the development of DNA methylation-based diagnostic and prognostic tools, as well as therapeutic interventions with drugs targeting the enzymes that catalyze DNA methylation reactions [7,8,9]. Research in the field of cancer epigenetics has clarified our understanding that global and gene-specific DNA methylation changes in somatic tissues often precede and might even induce the accumulation of cancer-related mutations [6,10]. Strong evidence supporting the notion that certain epigenetic modifications can be the first molecular event triggering the multi-step process of carcinogenesis is that constitutional epimutations, i.e., hypermethylation events occurring in utero, of tumor suppressor genes, such as *BRCA1, MLH1*, *MSH2*, and *MGMT*, have been observed in humans and linked to increased cancer risk [11,12]. Some of these constitutional epimutations are secondary events to cis-acting mutations in the proximity of the gene promoter region that induce their own methylation and silencing; however, others are primary epimutations observed in the absence of any genetic aberration and are likely induced by environmental factors [11,12,13]. Therefore, an emerging area of research is aimed at investigating the genetic and environmental factors that are able to induce the DNA methylation changes in somatic cells and tissues that potentially lead to cancer development and progression. In this article, I will comment on some recent articles describing the environmental factors linked to the aberrant DNA methylation levels of cancer-related genes.

Genetic and environmental factors may act during prenatal life, inducing epigenetic changes in the developing embryo and resulting in a constitutional epigenetic mosaicism that increases cancer risk in postnatal life [13]. However, exposure to environmental factors in postnatal life and adulthood can also induce epigenetic modifications in somatic tissues, leading to field effects capable of favoring the accumulation of somatic mutations and the development of cancer [6]. In a recent article published in *Cancers*, Ruiz de la Cruz and coworkers [13] reviewed the genetic and environmental factors that potentially lead to constitutional epigenetic mosaicism for cancer-related genes. Promoter hypermethylation results in gene silencing and has an effect similar to a loss of function mutation. The authors described several secondary epimutations resulting from cis-acting mutations in the 5′-untranslated region (5′-UTR) of tumor suppressor genes, such as c.-107A>T in *BRCA1*, c.-27C>A in *MLH1,* and c.-56C>T in *MGMT,* which are all linked to gene promoter hypermethylation, as well as *MSH2* hypermethylation resulting from a 5 Kb deletion at the 3′ end of the *EPCAM* gene located upstream of *MSH2* [13]. By contrast, primary epimutations occurring in the absence of any sequence variant are likely the result of environmental factors, including dietary factors, and exposure to heavy metals, cigarette smoke, endocrine-disrupting substances, air pollutants, and non-genotoxic carcinogens [13].

The link between tobacco smoking and cancer-related DNA methylation changes has recently been reviewed by Hoang and Landi [14] in the context of lung cancer. Indeed, tobacco smoking has been associated with DNA methylation changes in several lung cancer susceptibility genes, including *KLF6*, *STK32A*, *TERT*, *MSH5*, *ACTA2*, *GATA3*, *VTI1A*, and *CHRNA5*, suggesting that these genes might be regulated by methylation changes in response to smoking [15]. Similarly, it was observed that DNA methylation changes in certain CpGs at *AHRR*, 6p21.33, and *F2RL3* loci in blood DNA are associated with cigarette smoking and are predictive for lung cancer development [16]. Moreover, the hypermethylation of several cancer-related genes in lung cancer cells, including *CDKN2A, APC, MGMT, ANK1,* and *MTHFR*, has been associated with smoking [14]. A recent comparison of the DNA methylation signatures left by tobacco smoking between newborns from prenatal exposure and adults from personal smoking has been performed [17]. The study revealed thousands of differentially methylated CpGs that were unique to newborns in response to maternal smoking during pregnancy, along with many shared DNA methylation signatures by both newborns and adults that were related to either in utero or adulthood smoking exposure. Interestingly, the genes differentially methylated in relation to maternal smoking, but not to personal smoking in adults, were enriched in xenobiotic metabolism pathways. The study also revealed 2770 shared genes between newborns and adults, with the *AHRR* gene showing the highest number of differentially methylated CpGs [17]. Indeed, *AHRR* gene hypomethylation, in particular at cg05575921, is regarded as an epigenetic predictor of smoking status and intensity and could represent a potential blood biomarker to identify eligible smokers for lung cancer screening [18]. Interestingly, among non-smokers, hypomethylation of cg05575921 in the *AHRR* gene has been associated with particulate matter (PM_2.5_) exposure [19]. Notably, both tobacco smoking and PM_2.5_ exposure have been independently associated with *AHRR* cg05575921 hypomethylation in a dose-dependent manner [20].

Another interesting article by Gillman and coworkers [21] investigated the effects of aerobic exercise duration and intensity on the methylation levels of breast-cancer-related genes. The study hypothesis was based on evidence that exercise protects against cancer, and that certain changes in DNA methylation can precede breast cancer onset and might be the triggering event; for example, a primary somatic hypermethylation of *BRCA1* is not rare—it has been reported as a low-mosaic event in 4% to 10% of white blood cells from women across all age groups—and has been associated with increased risk of triple-negative breast cancer and high-grade serous ovarian cancer [12]. The authors selected women aged 30–45 years and evaluated the effects of 16 weeks of aerobic exercise of varying intensity and duration on the DNA methylation levels of a panel of breast-cancer-related genes in blood samples that were collected at the beginning of the exercise intervention and after its completion, as well as six months later at a follow-up visit. A total of 137 women, divided into four groups of different duration and intensity, completed the exercise intervention. Some genes, including *AURKA* and *BCAR1*, showed a linear slight increase in DNA methylation levels from baseline to follow-up, suggesting that exercise could have a long-lasting effect on their methylation levels. The total volume of exercise completion (duration + intensity) was not associated with the methylation levels of the investigated genes. However, a significant correlation was observed between an increase in maximal aerobic capacity (VO_2max_) over time and the decreased post-intervention methylation of *BRCA1.* The authors also observed that women that continued to regularly exercise during the six months from post-intervention to follow-up had lower levels of *GALNT9* methylation at follow-up [21]. Overall, the study by Gillman and coworkers provides preliminary evidence of some long-lasting effects of exercise on the DNA methylation patterns of breast-cancer-related genes [21]. Other environmental factors have been recently associated with altered *BRCA1* methylation levels. Examples include prenatal phthalate exposure, which was associated with increased *BRCA1* methylation in blood cord DNA [22]; intrauterine organochlorine pesticide exposure, which was associated with increased cord blood DNA methylation levels and reduced expression of *BRCA1* [23]; and serum levels of persistent organic pollutants, which have been associated with *BRCA1* methylation levels in men [24]. Breast cancer is the most common cancer observed in individuals exposed to the toxic dust and smoke resulting from the World Trade Center (WTC) towers attack on September 11, 2001, and a recent study assessed DNA methylation changes in blood DNA from WTC-exposed breast cancer patients, as well as WTC-exposed individuals who later developed breast cancer [25]. The study revealed global differences in DNA methylation levels and the increased methylation levels of several tumor suppressor genes in WTC-exposed individuals, including *BRCA1*, leading the authors to hypothesize that this could have resulted from exposure to the various toxic compounds present in dusts and fumes [25].

These are few examples of a broader recent literature linking environmental exposure to DNA methylation signatures potentially contributing to cancer development and progression. For example, recent evidence from genome-wide investigations in blood samples suggests that the analysis of DNA methylation signatures can improve the identification of asbestos-exposed individuals at higher risk to develop a malignant pleural mesothelioma [26,27]. Several studies [28] have investigated the epigenetic mechanisms in heavy-metal-induced carcinogenesis and angiogenesis. Indeed, there is growing evidence of cancer-related DNA methylation changes induced by chromium, nickel, and cadmium; however, most of the studies were carried out in cultured cells or xenograft models, with limited evidence in humans [28]. A recent study in children with acute lymphoblastic leukemia (ALL) revealed higher levels of organochlorine pesticides in ALL patients than in healthy matched controls. Moreover, the increase in organochlorine pesticide levels was associated with an increase in the promoter methylation levels of *CDKN2B* and *MGMT* coupled with global changes in histone tail modifications in ALL subjects, suggesting that exposure to organochlorine pesticides induces epigenetic changes, which may lead to ALL [29]. We recently reviewed the complex interactions among dietary factors and cancer-related epigenetic changes [30]. Several dietary factors are capable of regulating DNA methylation processes by acting as methyl donor compounds or as regulators of the activity of epigenetic enzymes. Indeed, diets enriched in saturated fatty acids and iron or depleted in protein and essential nutrients can induce global DNA methylation changes, ultimately promoting tumor aggressiveness. By contrast, many natural compounds, including folate, polyphenols, cinnamic acids, sulforaphane, and isothiocyanates, can exert a protective effect against cancer by acting as epigenetic modulators [30].

In summary, it is increasingly emerging in the literature that certain DNA methylation signatures left by human carcinogens, such as tobacco smoke, pesticides, asbestos, heavy metals, etc., often in a dose-dependent manner, could enhance our ability to stratify individuals according to their levels of exposure to dangerous risk factors and represent potential biomarkers to identify those at high risk to develop a particular cancer. Currently, polygenic risk scores (PRSs), which are based on combinations of inherited gene variants, are being increasingly investigated, often in conjunction with lifestyle factors, for their potential to estimate the individual risk of developing human cancers [31,32]. Unfortunately, it is often difficult to accurately measure past individual exposure to various environmental risk factors in personalized cancer risk assessment models. In this context, DNA methylation signatures could represent a valid tool to estimate past environmental exposures [33]. Therefore, combining PRSs and DNA methylation biomarkers is likely one of best strategies for a better understanding of gene–environment interactions in complex diseases. Furthermore, it could represent a valid tool to evaluate the benefits of dietary interventions, exercise, and lifestyle changes in cancer prevention strategies.
